# Effects of the ACE2-Ang-(1-7)-Mas axis on gut flora diversity and intestinal metabolites in SuHx mice

**DOI:** 10.3389/fmicb.2024.1412502

**Published:** 2024-08-23

**Authors:** Asimuguli Abudukeremu, Ainiwaer Aikemu, Tao Yang, Lei Fang, Adilai Aihemaitituoheti, Yupeng Zhang, Daliya Shanahaiti, Yiliyaer Nijiati

**Affiliations:** ^1^Central Laboratory of Xinjiang Medical University, Urumqi, China; ^2^College of Pharmacy, Xinjiang Medical University, Urumqi, China; ^3^Department of Pharmacy, College of Xinjiang Uyghur Medicine, Hetian, China; ^4^Xinjiang Key Laboratory of Hetian Characteristic Chinese Traditional Medicine Research, Hetian, China; ^5^Engineering Research Center for Quality Control of Uyghur Medicinal Materials and Preparations, Hetian, China; ^6^Key Laboratory of High Incidence Disease Research in Xinjiang (Xinjiang Medical University), Ministry of Education, Urumqi, China

**Keywords:** SuHx, ACE2-Ang-(1-7)-Mas, gut flora diversity, metabolomics, correlative analyses

## Abstract

**Objective:**

Pulmonary artery hypertension (PAH) poses a significant challenge due to its limited therapeutic options and high mortality rates. The ACE2-Ang-(1-7)-Mas axis plays a pivotal role in regulating blood pressure and inhibiting myocardial remodeling. However, the precise mechanistic links between the ACE2-Ang-(1-7)-Mas axis and PAH remain poorly understood. This study aimed to elucidate the involvement of the ACE2-Ang-(1-7)-Mas axis in the development of PAH.

**Methods:**

PAH was induced in mice using Sugen5416/hypoxia, PAAT/PET ratio and PA were detected using cardiac ultrasound; inflammation related factors such as MCP-1, TNF, IL-10and IL-12p70 were detected in intestines using cytometric bead array (CBA) kits; histopathological and morphological changes in lung and intestinal tissues were assessed via HE staining and Masson staining to evaluate the progression of PAH. Immunohistochemistry and western blotting were employed to determine the expression levels of two tight junction proteins, occludin and ZO-1, in intestinal tissues. Additionally, 16rRNA sequencing and non-targeted metabolomics by LC-MS/MS techniques were utilized to investigate the impact of the ACE2-Ang-(1-7)-Mas axis on microbial diversity and metabolomics of intestinal contents.

**Results:**

Activation of the ACE2-Ang-(1-7)-Mas axis improves heart function, reduces intestines inflammatory factors and ameliorates pathological and histological alterations in SuHx mice. This activation notably upregulated the expression of occludin and ZO-1 proteins in intestinal tissues and promoted the proliferation of SCFA-producing bacteria genera, such as *g_Candidatus_Saccharimonas*. Furthermore, it enhanced the abundance of beneficial metabolites, including tryptophan and butyric acid.

**Conclusion:**

The findings suggest that modulation of the ACE2-Ang-(1-7)-Mas axis can alleviate PAH by regulating intestinal microbes and metabolites. These results highlight the potential of the ACE2-Ang-(1-7)-Mas axis as a promising therapeutic target for clinical management of PAH.

## Graphical abstract

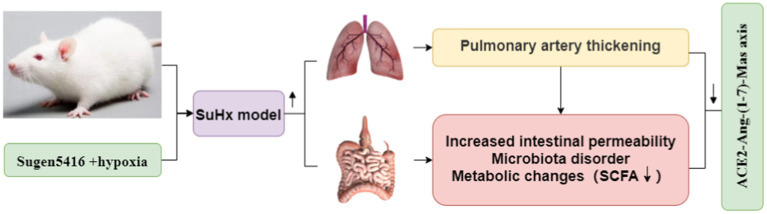

## Introduction

Pulmonary arterial hypertension (PAH) represents a severe clinical condition characterized by persistent vasoconstriction, vascular remodeling, perivascular inflammation, and fibrinoid necrosis. These pathological changes result in the narrowing and occlusion of the vascular lumen, leading to a progressive elevation in pulmonary vascular resistance and eventual right heart failure (Guignabert and Dorfmuller, 2013; Poch and Mandel, 2021). Notably, Sugen5416, by specifically binding to endothelial cells, triggers endothelial cell proliferation via sustained inhibition of vascular endothelial growth factor receptors, culminating in the formation of occlusive plexiform lesions (Neto-Neves et al., 2017). In the Sugen5416/hypoxia-induced pulmonary hypertension model, a hallmark histological feature is the pronounced hypertrophy and proliferation of pulmonary artery endothelial cells and pulmonary vascular smooth muscle cells. Unlike the chronic hypoxia alone model, the lesions observed in the Sugen5416/hypoxia (SuHx) model are irreversible (de Raaf et al., 2014). Despite continuous efforts and innovative strategies aimed at developing effective treatments, the options to impede the progression of pulmonary hypertension remain limited, rendering it a fatal disease.

The renin-angiotensin system (RAS) is integral to maintaining cardiovascular homeostasis and regulating water and electrolyte balance in mammals. Additionally, it plays key roles in cellular processes such as proliferation, differentiation, and apoptosis (Forrester et al., 2018). Of particular significance within the RAS are the antagonistic ACE-AngII-AT1R axis and the coordinating ACE2-Ang-(1-7)-Mas axis, wherein angiotensin converting enzyme 2 (ACE2) serves as an isoenzyme of angiotensin converting enzyme (ACE) (Shenoy et al., 2010), ACE2 counteracts the effects of ACE by promoting the synthesis of angiotensin (1-7) (Ang-(1-7)) and degrading angiotensin II (AngII) (Zhang et al., 2021). Chronic hypoxia induces an imbalance between ACE and ACE2 via hypoxia inducible factor-1α (HIF-1α), resulting in increased ACE levels and decreased ACE2 expression (Sharma et al., 2020a,b). ACE-mediated conversion of AngI to AngII leads to degradation of vasodilatory substances, thereby inducing pulmonary vasoconstriction, elevating pulmonary vascular resistance, and promoting proliferation of pulmonary arterial smooth muscle, vascular remodeling, and pulmonary hypertension. Studies utilizing ACE2 knockout mice have demonstrated abnormal cardiac development and severely impaired cardiac contractility, while ACE2 overexpression in the heart has shown protective effects against myocardial injury (Dang et al., 2020). Notably, an ACE2 receptor agonist, XNT, has been found to prevent the development of pulmonary hypertension and mitigate associated damage (Gheblawi et al., 2020). The ACE2-Ang-(1-7)-Mas axis exhibits multifaceted biological effects, including negative regulation of blood pressure, inhibition of myocardial remodeling, reduction of inflammatory factor production, and counteraction of the adverse effects mediated by the ACE-AngII-AT1R axis. These effects collectively suggest potential benefits for PAH pathophysiology (Zhang et al., 2021).

The intestinal flora, comprising a vast array of microorganisms inhabiting the animal intestine, serves as crucial companions to host cells and an integral aspect of precision medicine. These microbes play a pivotal role in the absorption and metabolism of both exogenous and endogenous substances. Consequently, various components of the intestinal microbiota and their metabolites can elicit diverse biological effects. With ongoing advancements in intestinal flora research, its potential in disease intervention has become increasingly evident (Chen et al., 2022). The gut microbiota constitutes a diverse community of microorganisms residing in gastrointestinal tissues, closely interacting with the host to provide genetic, metabolic, and immune benefits (Lozupone et al., 2012), alterations in gut microbiota composition and the activation of inflammation suggest the involvement of gut ecological dysregulation in the inflammatory processes associated with PAH. Dysbiosis, characterized by an imbalance in the gut microbial community, has been observed in models of SuHx induced and monocrotaline induced pulmonary hypertension (PH) (Callejo et al., 2018; Sharma et al., 2020a,b). Exposure to low-pressure hypoxia can induce gastrointestinal injury, oxidative stress, and increased gastrointestinal permeability, accompanied by changes in gut microbial composition and activity (Adak et al., 2014). Hypoxia exposure reduces oxygen pressure in the small intestine, further influencing gut microbiota composition. Activation of the sympathetic nervous system in SuHx rats may increase intestinal permeability, contributing to ecological dysregulation. Compared to controls, SuHx rats exhibited increased abundance of 14 genera of bacteria, including *Bacteroides* and *Akkermansia*, while the abundance of 7 genera of bacteria, including *Rothia* and *Prevotellaceae* was decreased (Sanada et al., 2020). In a study involving ACE2KI-N mice, Chao1 abundance, Shannon diversity, and homogeneity were significantly higher compared to WT-N mice, resulting in a lower F/B ratio. Intestinal ACE2 activity was notably higher in the ACE2KI-N and ACE2KI-H groups, although hypoxia decreased intestinal ACE2 activity in the ACE2KI-H group compared to the ACE2KI-N group. Full-scale overexpression of ACE2 rendered the animals resistant to hypoxia-induced intestinal pathology, alterations in microbiota, and neuroinflammation. Overall, ACE2 overexpression protected mice from neuroinflammation, microbiota alterations, and PH-related intestinal pathology. Intestinal repopulation with fecal microbiota from ACE2KI mice mitigated the hypoxia-induced increase in right ventricular systolic pressure (RVSP) and right ventricular hypertrophy in WT mice (Sharma et al., 2020a,b).

Emerging research underscores the significant impact of intestinal flora dysbiosis on the host’s pathophysiological state, mediated through various mechanisms including immune and metabolic alterations. Dysbiosis, characterized by an imbalance in intestinal flora, can foster the proliferation of potentially pathogenic bacteria, bacterial translocation, and release of endotoxins, thereby elevating intestinal epithelial reactive oxygen species (ROS) levels and increasing intestinal permeability (Ailizire et al., 2023). Fecal metabolites serve as reflective indicators of the intestinal flora state and the symbiotic relationship between flora and the host. Combining fecal metabolomics with 16SrRNA gene sequencing offers a comprehensive approach to elucidating the intricate interplay between gut microbiota and the host. PAH poses a significant challenge due to its limited therapeutic options and high mortality rates. The ACE2-Ang-(1-7)-Mas axis plays a pivotal role in regulating blood pressure and inhibiting myocardial remodeling. However, the precise mechanistic links between the ACE2-Ang-(1-7)-Mas axis and PAH remain poorly understood. In this paper, the findings suggest that modulation of the ACE2-Ang-(1-7)-Mas axis can alleviate PAH by regulating intestinalmicrobes and metabolites. These results highlight the potential of the ACE2-Ang-(1-7)-Mas axis as a promising therapeutic target for clinical management of PAH. Therefore, the objective of this study was to establish a chronic SuHx mouse model within a hypoxia chamber to investigate its impact on intestinal injury, gut microbiota characteristics, and fecal metabolomics (Zhou et al., 2020).

## Materials and methods

### SuHx animal model establishment and pharmacological intervention

**Figure 1:**
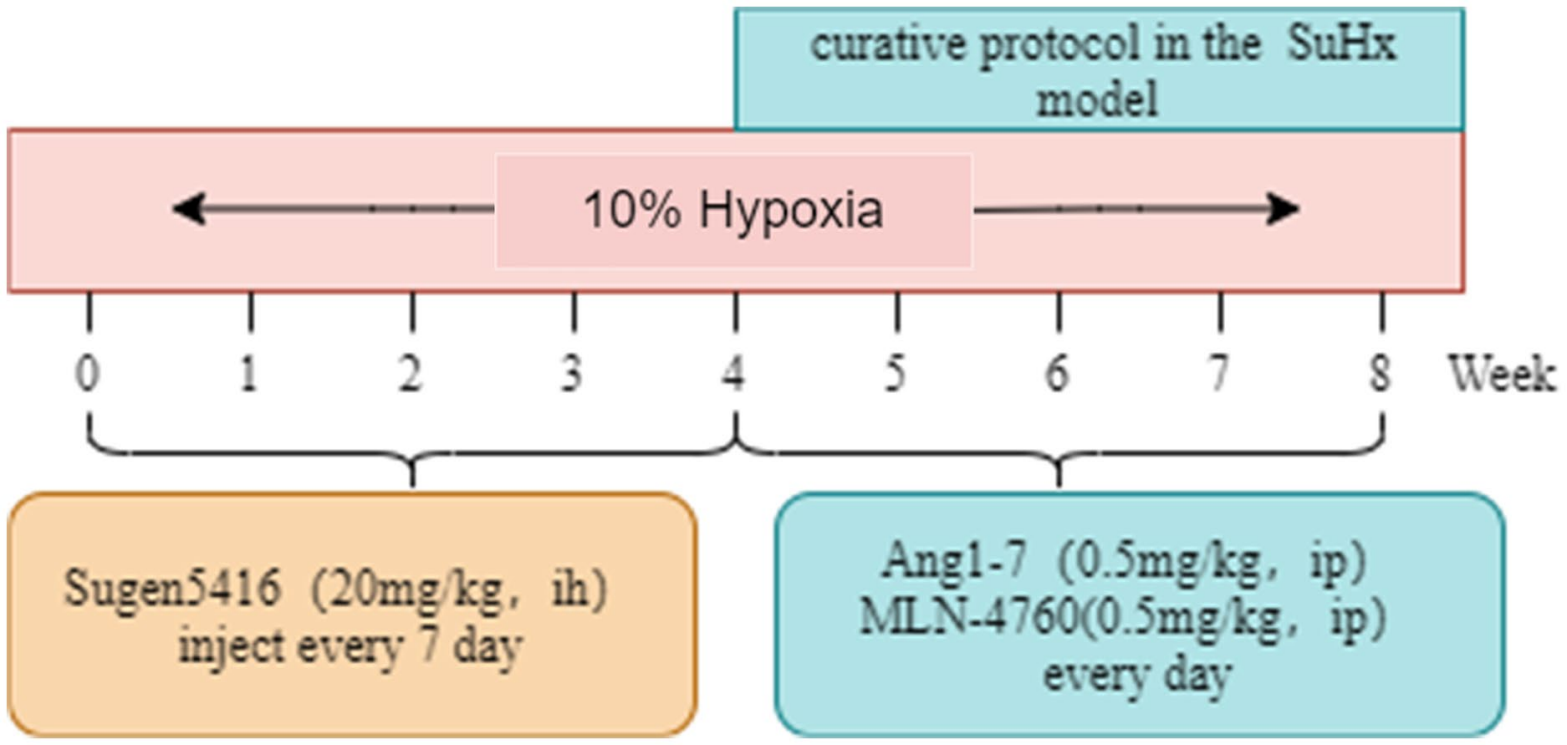
Experiment protocol.

Sixty 6-week-old male BALB/C mice were housed in a controlled environment with a temperature of 22°C and humidity of 50%, under a 12 h light/dark cycle. They had *ad libitum* access to water and food throughout the study. The control group (Con) consisted of mice housed at an altitude of 800 m. To induce the Sugen5416/hypoxia (SuHx) model, mice were subcutaneously injected with 20 mg/kg of Sugen5416 (MCE, Cat# HY-10374) once a week for 4 weeks under hypoxia conditions (10% O_2_). Following Sugen5416 administration, the mice were exposed to chronic hypoxia (10% O_2_) for an additional 4 weeks. After 4 weeks, the mice rats were divided into 4 groups: (A) Control group (Con): on the basis of Con group, saline was given for the last 4 weeks and injected intraperitoneally. (B) SuHx group (SuHx): on the basis of SuHx group, saline was given for the last 4 weeks and injected intraperitoneally. (C) SuHx + Ang1-7 intervention group (SuHxA): on the basis of SuHx group, Ang1-7 (0.5 mg/kg, MCE, Cat# HY-12403) was given for the last 4 weeks and intraperitoneal injection. (D) SuHx + MLN4760 group (SuHxM): on the basis of SuHx group, MLN-4760 (0.5 mg/kg, MCE, Cat# HY-19414), an ACE2 inhibitor, was given intraperitoneally on the latter 4 weeks (Figure 1).

### Echocardiography

The mice were anesthetized with 1% sodium pentobarbital (45 mg/kg) by intraperitoneal injection, and the breast region was prepared. The structural changes on the heart of rats in each group were investigated by Doppler color ultrasound, including the pulmonary artery blood flow acceleration time (PAAT), pulmonary artery ejection time (PET), pulmonary artery inner diameter (PA) and structural changes, etc., the cardiac motion state was observed, and the relevant parameters were measured and recorded.

### Pathological examination

#### Hematoxylin-eosin staining

Intestinal and lung tissues were excised and immediately rinsed in saline, followed by fixation in 4% formaldehyde for 24 h. Subsequently, the tissues were washed in PBS and embedded in paraffin. Sections of 4 μm thickness were cut, baked, dewaxed, and dehydrated using gradient ethanol. Hematoxylin staining was performed for 5 min, followed by differentiation in 1% ethanol solution for 5 s and 1% ammonia solution for 30 s to restore the blue color. Eosin staining solution was applied for 1 min, followed by sequential immersion in 80% ethanol, 95% ethanol I, 95% ethanol II, anhydrous ethanol, xylene I, and xylene II for 5 s each. The sections were then sealed, air-dried, and observed under a microscope for image capture.

#### Masson staining

Sections of intestinal tissue were prepared, deparaffinized, and dehydrated. Hematoxylin staining was performed for 5 min, followed by differentiation in acidic ethanol differentiation solution for 8 s. Blueing solution was applied to restore the blue color, followed by staining with Lichun red magenta. Subsequently, the sections were washed with weak acid working solution and phosphomolybdic acid solution for 1.5 min each. Aniline blue was applied for 1 min, followed by rinsing with distilled water to stop the staining process. The sections were then dehydrated in 95% ethanol for 2 s, followed by three rounds of differentiation in anhydrous ethanol for 10 s each and xylene for 1 min each. Finally, the sections were sealed, dried, observed, and photographed.

#### Immunohistochemistry

Paraffin sections of lung tissue and intestinal tissue were defatted and rehydrated. Antigen retrieval was performed by placing the sections in citrate buffer under high pressure and heat. After washing with PBS, the sections were incubated with 3% hydrogen peroxide for 15 min to remove endogenous catalase. Subsequently, the sections were incubated in goat serum solution at room temperature in the dark for 20 min, followed by incubation with primary antibodies against α-smooth muscle actin (α-SMA) (Affinity, Cat# AF1032, 1:400), ZO-1 (Proteintech, Cat# 21773-1-AP, 1:3,000) and occludin (Proteintech, Cat# 27260-1-AP, 1:5,000) overnight at 4°C. After washing with PBS, the sections were incubated with secondary antibody solution in a 37°C water bath for 20 min. The sections were then stained with DAB to visualize positive staining, followed by counterstaining with hematoxylin and routine sealing. Staining results were observed and photographed under a light microscope, and the positive expression rates of α-SMA, ZO-1 and occludin were calculated based on the number of positive nuclei (brown-yellow staining) relative to the total number of nuclei.

### Cytometric bead array assay

Take 30 mg intestinal tissue, add 180 μL PBS containing protease inhibitor to grind, centrifuge 500 × g for 10 min, and take supernatant for detection. Fifty microliters of intestines sample was gently mixed with an equal volume of the mixed captured beads (BD, Cat# 552364). All assay tubes were added 50 μL of the PE detection reagent and incubated in the dark at room temperature for 2 h. After the incubation, each assay tube was added 1 mL of Wash Buffer and centrifuged at 200 g for 5 min, followed by discarding the supernatant. The bead pellet was resuspended with 300 μL of Wash Buffer and all samples were tested by flow cytometry (BD, LSR Fortessa).

### Western blotting analysis

Intestinal tissue was retrieved for grinding and homogenizing. Proteins were extracted, and their concentration was determined using the BCA method. Each sample’s concentration was then normalized using lysate. A 7.5% SDS-PAGE gel was prepared, and the protein was loaded as a top sample for gel electrophoresis to separate the proteins. Subsequently, the membranes were subjected to a constant flow rate of 100 V, allowing the separated target proteins to be transferred onto PVDF membranes. These membranes were then incubated with 5% skimmed milk powder sealing solution for 2 h. Following this, the membranes were separately immersed in 1xTBST diluted with ZO-1 (Affinity, Cat# AF1032, 1:1,000) and occludin (Proteintech, Cat# 27260-1-AP, 1:1,000) monoclonal antibody working solutions overnight at 4°C. The next day, the membranes were washed three times with 1xTBST for 10 min each, followed by co-incubation with horseradish peroxidase-labelled goat anti-rabbit IgG (1:20,000) diluted in 1xTBST for 1 h at room temperature. Subsequently, the membranes were washed again with 1xTBST. The images were developed in a dark box using enhanced ECL chromogenic solution, and captured using a gel imaging system. The grey value of each target protein and the internal reference protein actin were analyzed using Image-Pro Plus 6.0 software. The ratio of the grey value of the target protein to that of the internal reference protein was considered as the relative expression of the target proteins.

### 16SrRNA sequencing analysis of intestinal contents

The mice were anesthetized and euthanized, and the specimens were aseptically removed in a sterile environment. Approximately 0.3 g of intestinal contents were collected and placed in sterilized centrifuge tubes. The samples were rapidly frozen in liquid nitrogen and then stored at −80°C. Total genomic DNA extraction of the microbial community was performed according to the instructions of the E.Z.N.A.^®^ soil DNA kit (Omega Bio-tek, Norcross, GA, United States). Using the extracted DNA as a template, the V3–V4 variable region of the 16SrRNA gene was amplified via PCR with the forward primer 338F (5′-ACTCCTACGGGGAGGCAGCAG-3′), which included the Barcode sequence, and the reverse primer 806R (5′-GGACTACHVGGGTWTCTAAT-3′). The PCR reaction system consisted of 4 μL of 5× TransStart FastPfu buffer, 2 μL of 2.5 mM dNTPs, 0.8 μL of the forward primer (5 μM), 0.8 μL of the reverse primer (5 μM), 0.4 μL of TransStart FastPfu DNA polymerase, 0.2 μL of BSA, and 10 ng of template DNA, with the final volume adjusted to 20 μL. The amplification procedure included an initial denaturation step at 95°C for 3 min, followed by 27 cycles of denaturation at 95°C for 30 s, annealing at 55°C for 30 s, and extension at 72°C for 45 s, and a final extension at 72°C for 10 min. The PCR products were then stored at 4°C. Purified PCR products were used for library construction with the NEXTFLEX Rapid DNA-Seq Kit and quantified using Quantifluor-ST (Promega, Madison, WI, United States). Sequencing was performed according to standard procedures on the Illumina MiSeq platform by Major Biomedical Technology Co.

### Metabolomics analysis of gut contents

The mice were anesthetized and euthanized, and the specimens were aseptically removed in a sterile environment. Approximately 0.3 g of intestinal contents were collected and placed in sterilized centrifuge tubes. The samples were rapidly frozen in liquid nitrogen and then stored at −80°C. Non-targeted metabolomics analyses were conducted using LC-MS/MS technology. Positive and negative ion data were collected using an ultra-high performance liquid chromatography tandem time-of-flight mass spectrometry (UPLC-TripleTOF) system (AB SCIEX) to enhance metabolite coverage. Progenesis QI software (Waters Corporation, Milford, United States) was utilized for LC-MS/MS data processing, which included peak extraction, peak comparison, and compound identification. Pre-processed data were uploaded to the Meggie Bio cloud platform^1^ for further analysis. The R package ropls (Version 1.6.2) was employed to perform principal component analysis (PCA) and orthogonal least partial squares discriminant analysis (OPLS-DA). Model stability was assessed using seven cycles of cross-validation. Student’s *t*-test and multiplicative analysis of variance were conducted. Significantly different metabolites were selected based on variable weight values (VIP) obtained from the OPLS-DA model and the student’s *t*-test *p*-value. Metabolites with VIP >1 and *p* < 0.05 were considered as significantly different metabolites. A total of 1,779 and 1,496 differential metabolites were identified in electrospray ionization positive (ESI+) mode and electrospray ionization negative (ESI−) mode, respectively. Pathway analysis of the differential metabolites was performed using the KEGG database. Pathway enrichment analysis was conducted using the Python package, scipy.stats, and Fisher’s exact test to determine the most relevant biological pathways associated with the experimental treatments.

### Statistical analyses

All data analyses were conducted using the Meggie BioCloud platform (see text footnote 1). Results were presented as mean ± standard deviation (SD). Statistical analyses were carried out using the unpaired Student’s t-test for comparisons between two groups or ANOVA for comparisons among multiple groups. Software employing the Wilcoxon test or Kruskal–Wallis test was utilized to determine the significance of genera or functions. Statistical significance was denoted as follows: ^*^p < 0.05, ^**^p < 0.01, and ^***^p < 0.001 vs. SuHx group.

## Results

### Effects of the ACE2-Ang-(1-7)-Mas axis on Sugen5416/hypoxia induced PAH mice

**Figure 2:**
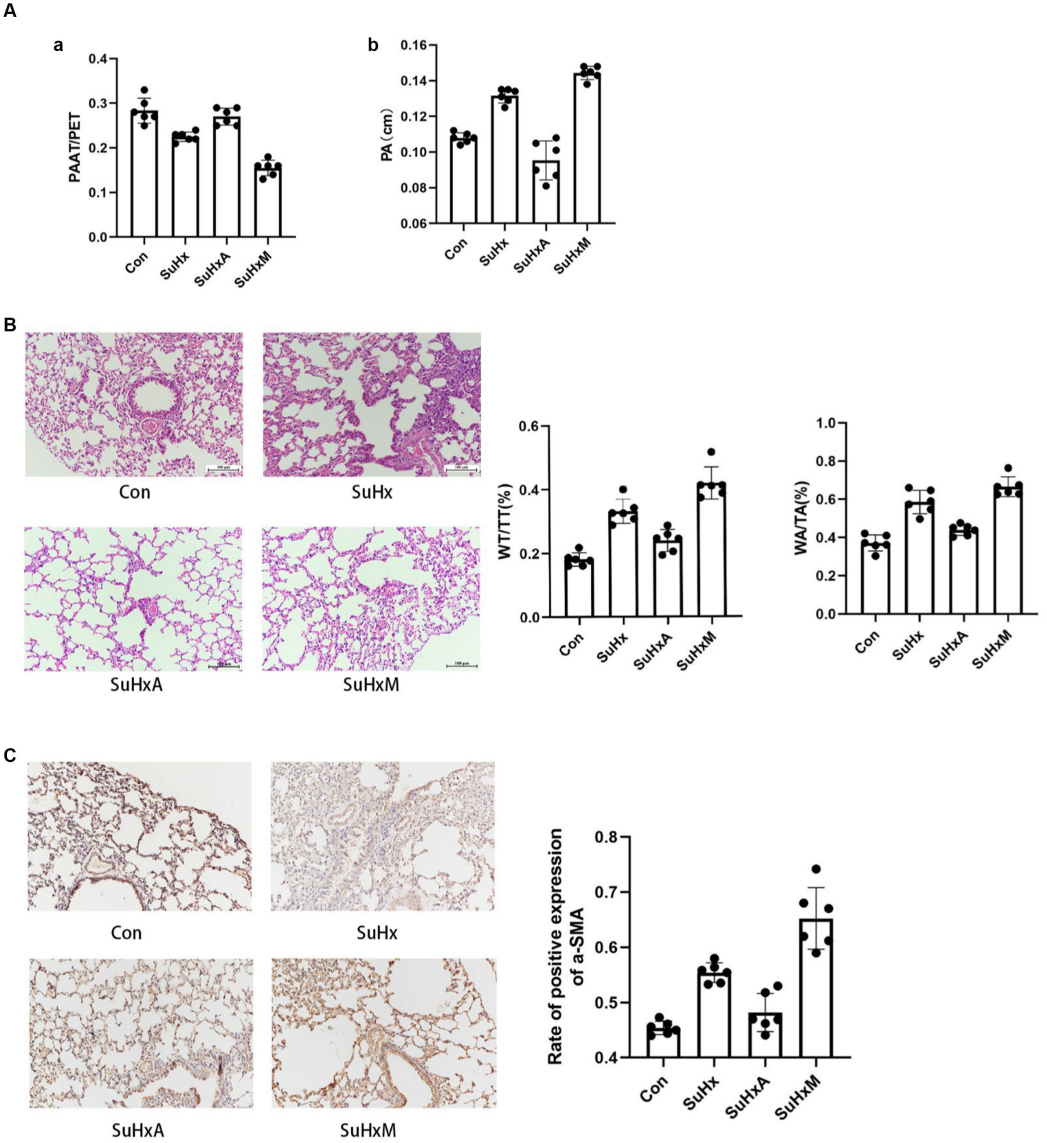
Sugen5416/hypoxia induced PAH mouse results. **(A)** Histogram of PAAT/PET ratio and PA bar chart. **(B)** Typical HE staining of small pulmonary arteries in the peripheral lungs as well as histograms of wall area/total area of the small pulmonary arteries (WA/TA) and wall thickness/total thickness of the small pulmonary arteries (WT/TT). **(C)** Immunohistochemical detection of α-SMA expression levels in lung tissue. *N* = 6 in each group.

The Echocardiography results showed that the PAAT/PET ratio was reduced and the PA widened in the SuHx mice compared with the control group; The PAAT/PET ratio and PA were basically restored to normal levels after Ang1-7 treatment compared with the SuHx group, but the reduction in the PAAT/PET ratio and the widening of the PA were more pronounced after MLN-4760 intervention (Figure 2A).

Histological examination via hematoxylin-eosin (HE) staining revealed a noteworthy thickening of the pulmonary artery wall and luminal narrowing in SuHx mice compared to control mice. Notably, treatment with Ang1-7 led to a gradual improvement in these abnormalities. Conversely, treatment with MLN-4760 resulted in a significant exacerbation of these phenomena compared to the SuHx group (Figure 2B). In addition, immunohistochemistry of lung tissue showed the degree of mesangial smooth muscle hypertrophy and hyperplasia as reflected by the expression level of α-SMA. The expression level of α-SMA was significantly increased in SuHx group mice compared with the control group, and decreased after intervention with Ang1-7, but abnormally increased after intervention with MLN-4760 (Figure 2C).

### Effect of ACE2-Ang-(1-7)-Mas axis on intestinal function in SuHx mice

**Figure 3:**
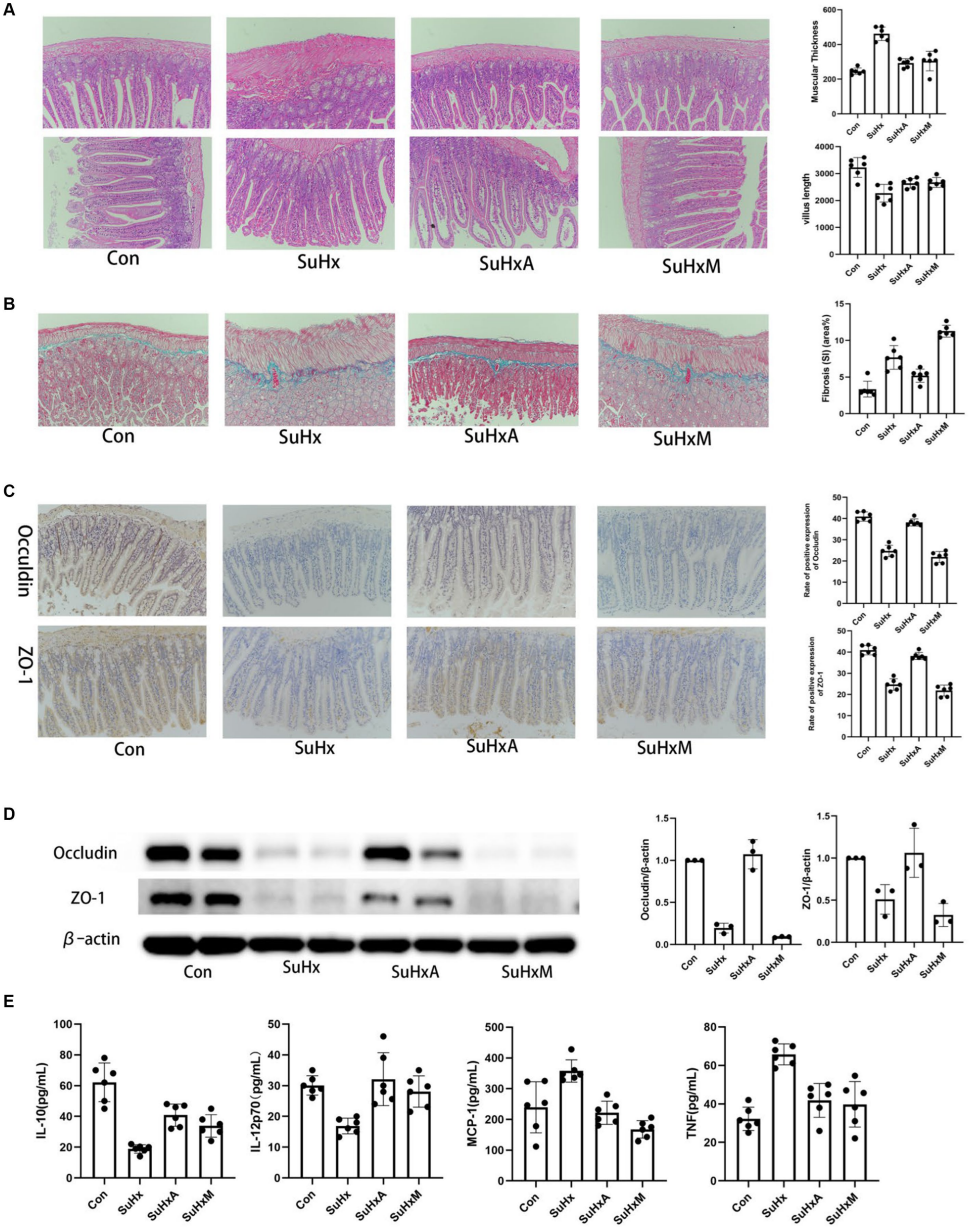
Morphological changes and protein expression levels in the intestines of mice in each group. **(A)** Intestinal tissue HE staining graphs, intestinal tissue muscularis propria thickness and chorionic villus length histograms. **(B)** Masson staining of intestinal tissues stained with collagen staining in blue. **(C)** Intestinal tissue occludin and ZO-1 positive expression rate. **(D)** Intestinal tissue occludin and ZO-1 protein expression level. **(E)** Statistical chart of inflammation-related factor levels. *N* = 6 in each group.

Histological examination of the intestinal tract by HE staining and Masson staining showed that the (Figures 3A,B) demonstrated a significant increase in intestinal fibrosis and muscle thickness, coupled with a decrease in villi length, in SuHx group mice compared to control mice. Conversely, treatment with Ang1-7 led to a significant reduction in intestinal fibrosis and muscle thickness, along with an increase in villi length. These findings underscore the pivotal role of an intact intestinal barrier in maintaining organismal homeostasis.

Impairment of the intestinal mucosal barrier function can heighten host susceptibility to intestinal antigens and pathogens, potentially disrupting immune homeostasis and inciting inflammatory responses. The formation of epithelial tight junctions (TJs) is crucial for maintaining the integrity of the intestinal barrier. TJs typically consist of proteins such as occludin and ZO-1, which are indispensable for epithelial barrier integrity. Immunohistochemistry and protein immunoblotting of intestinal tissues revealed decreased expression of TJ proteins occludin and ZO-1 in the SuHx and SuHxM groups, indicative of increased intestinal permeability in these mice. Notably, treatment with Ang1-7 led to an augmentation in the expression of occludin and ZO-1, thereby ameliorating intestinal permeability (Figures 3C,D).

Inflammatory factors levels have been shown to be an independent predictor of survival in patients with PH and, in combination with other markers of disease severity, can predict patient prognosis (Heresi et al., 2014). The results of the cytometric bead array (CBA) kit showed that the levels of pro-inflammatory factor such as monocyte chemotactic protein-1 (MCP-1) and tumor necrosis factor (TNF) and were significantly elevated but anti-inflammatory factor such as interleukin-10 (IL-10) and interleukin 12p70 (IL-12p70) were reduced in the SuHx group compared to the control group. Ang1-7 and MLN-4760 treatments resulted in a progression of these cytokine’s towards normal levels (Figure 3E).

### Effect of ACE2-Ang-(1-7)-Mas axis on gut microbiological changes in SuHx mice

**Figure 4:**
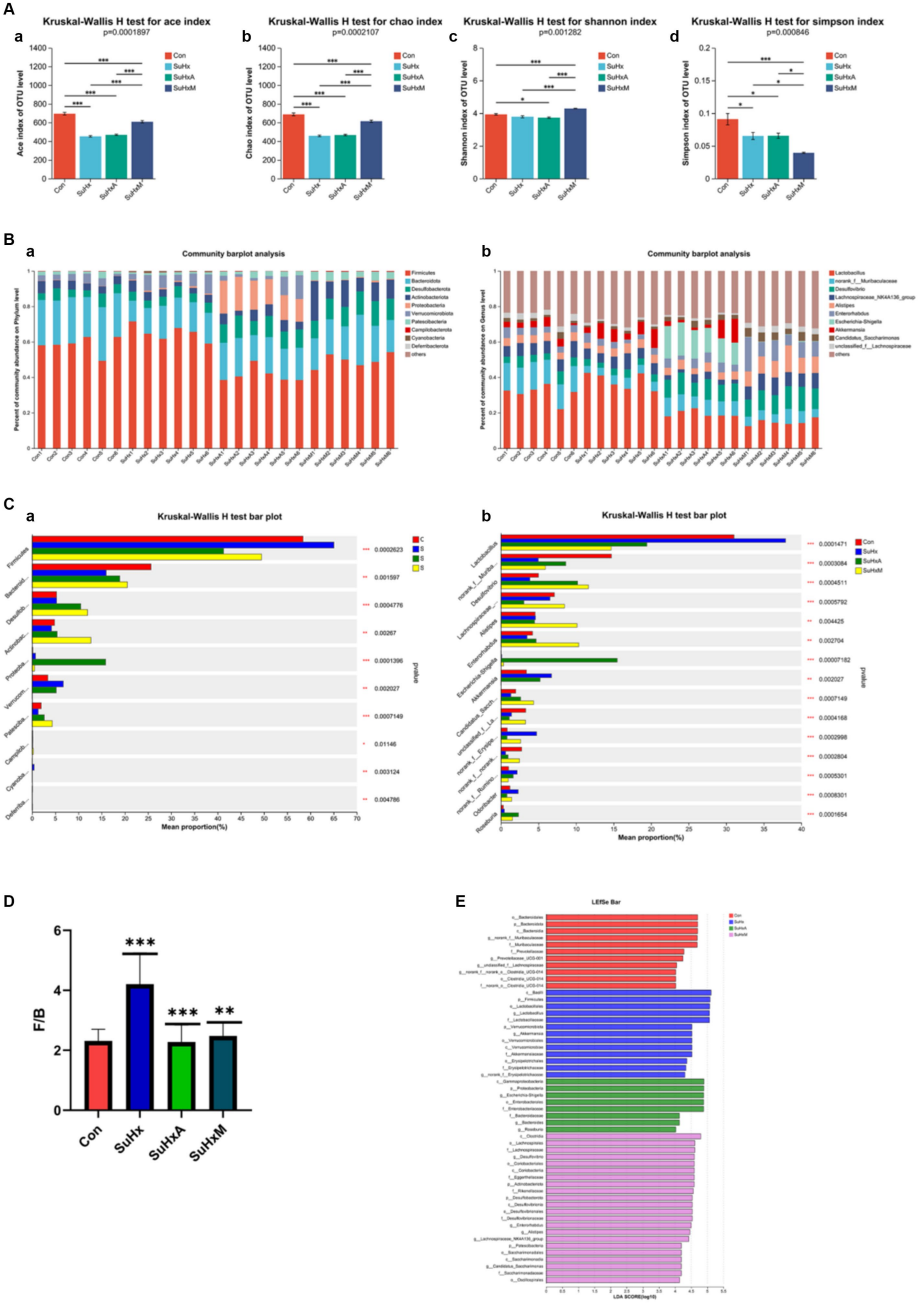
Alterations in the gut microbiome of mice in each group. **(A)** Alpha diversity of mice in each group. **(a–d)** Ace, Chao, Shannon, Simpson indices. **(B,a,b)** Gut microbiome composition at the family and genus level, respectively. The relative abundance of microbiota below 0.5% in all samples was combined as others in the histograms. **(C,a,b)** Analysis of species differences between multiple groups at the family and genus levels, respectively. **(D)** F/B ratio for each group. **(E)** LEfSe for each group to show differences in bacterial genera. *N* = 6 in each group.

Initially, α-diversity analysis revealed significant differences, with notable variations observed in the Ace and Chao indices among the groups, indicating alterations in bacterial species abundance and community richness induced by Sugen5416/hypoxia (Figure 4A).

Secondly, at the portal level, the gut microbiota was mainly composed of Firmicutes, Bacteroidota, Desulfobacterota, Actinobacteriota, Proteobacteria, Verrucomicrobiota, and Patescibacteria (Figure 4B,a), with Firmicutes having the highest abundance, followed by *Bacteroidota*, *Desulfobacterota* and *Actinobacteriota*, among others. At the genus level, it consisted of *Lactobacillus*, *norank_f_Muribaculaceae*, *Desulfovibri*, *Lachnospiraceae_NK4A136_group*, *Alistipes*, *Enterorhabdus*, *Enterorhabdus* and *Akkermansia* et al. (Figure 4B,b). The *Firmicutes*/*Bacteroidetes* (F/B) ratio, a marker of intestinal dysfunction in hypertensive patients, was elevated in the SuHx group compared to control mice. Notably, treatment with Ang1-7 and MLN-4760 restored the F/B ratio to near-normal levels (Figure 4D).

Subsequently, significant differences were observed in specific bacterial genera between the control, SuHx, SuHxA and SuHxM treatments. Specifically the microbial phylum *Firmicutes* (*p* = 0.0002623) was significantly more in the SuHx group and significantly less in the SuHxA and SuHxM groups, while the microorganisms *Bacteroidota* (*p* = 0.001597), *Actinobacteriota* (*p* = 0.00267), *Patescibacteria* (*p* = 0.0007149) and other microorganisms were significantly reduced in SuHx group and significantly increased in SuHxA and SuHxM groups (Figure 4C,a). At the level of microbial genera, *norank_f_Muribaculaceae* (*p* = 0.0003084), *Enterorhabdus* (*p* = 0.002704) and *Candidatus_Saccharimonas* (*p* = 0.0007149) were significantly reduced in SuHx group and significantly increased in SuHxA and SuHxM groups were significantly increased (Figure 4C,b), while harmful genera such as *norank_f_Erysipelotrichaceae* (*p* = 0.0002998) were significantly increased in the SuHx group. In addition, linear discriminant analysis of effective size (LEfSe) was used to account for differences in microbial genera, and these findings collectively showed alterations in the gut microbiomes of the control, SuHx, SuHxA, and SuHxM groups (Figure 4E).

### Effect of ACE2-Ang-(1-7)-Mas axis on intestinal metabolites in SuHx mice

**Figure 5:**
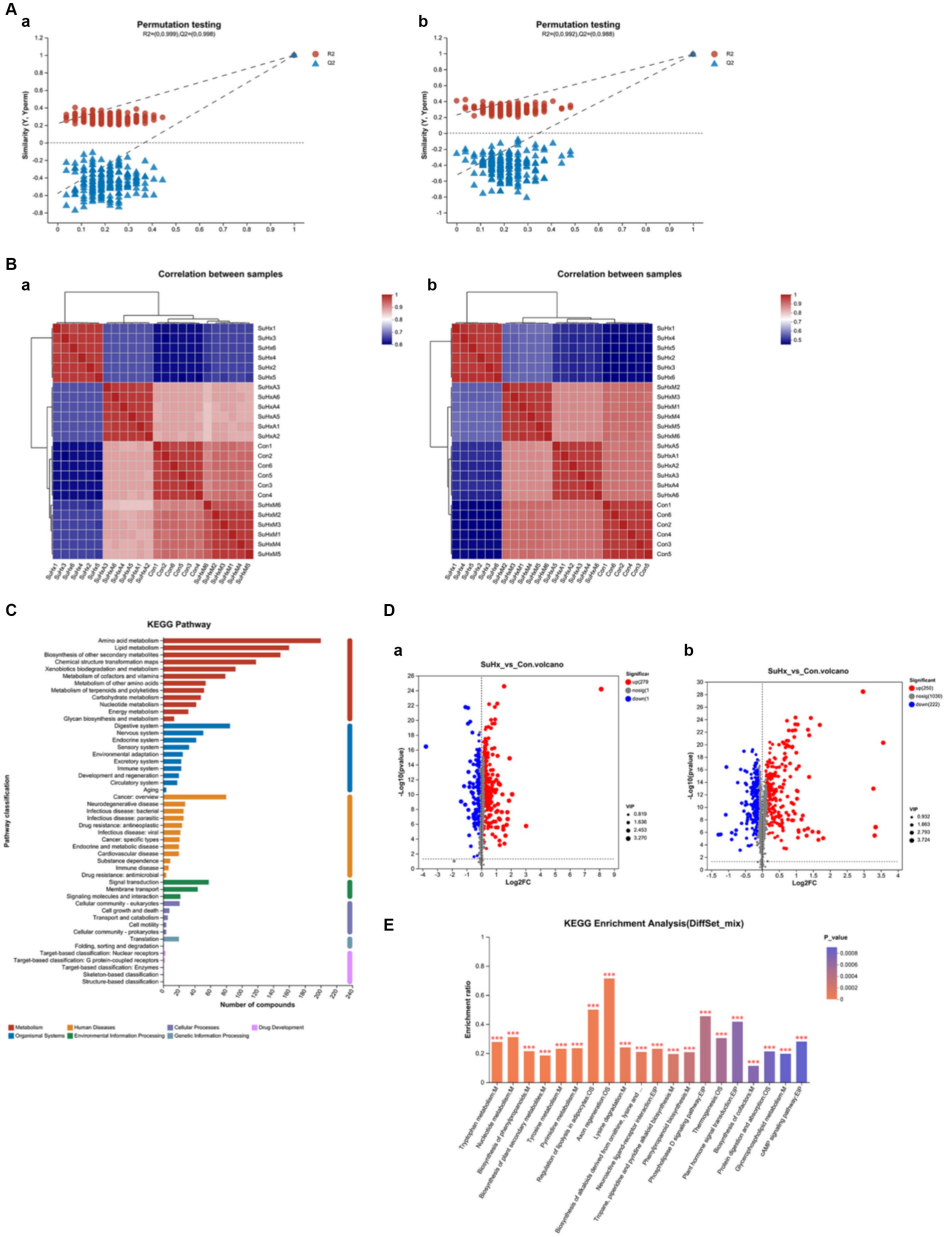
Changes in the metabolome of mouse intestinal contents in each group. **(A,a,b)** Validation plots of the PLS-DA model based on serum metabolome in electrospray ionised positive ions (ESI+) **(a)** and electrospray ionised negative ions (ESI−) **(b)**. **(B,a,b)** Heatmaps based on metabolic profiles of gut contents in ESI+ **(a)** and ESI− **(b)**. **(C)** KEGG function annotation bar graph, *X*-axis indicates the number of metabolite annotations and *Y*-axis indicates the annotated KEGG pathways. **(D,a,b)** Volcano plots based on the metabolic profiles of intestinal contents in the SuHx group vs. control group in electrospray ionised positive ions (ESI+) **(a)** and electrospray ionised negative ions (ESI−) **(b)**. The volcano plots show the differential abundance of gut content metabolites (VIP >1 or *p*-value <0.05). Red dots represent a differential increase in metabolites, and blue dots represent a differential decrease in metabolites. **(E)** KEGG enrichment analysis plots, with the horizontal coordinate indicating the pathway name and the vertical coordinate indicating the enrichment rate; *N* = 6 in each group.

To further elucidate the intestinal metabolite profile of the ACE2-Ang-(1-7)-Mas axis in SuHx mice, the intestinal contents of our groups of mice were subjected to LC/MS non-targeted metabolic profiling in both positive and negative modes. In electrospray ionisation positive (ESI+) mode and electrospray ionisation negative (ESI−) mode, 1,779 and 1,496 metabolites were detected in the intestinal contents, respectively, and 1,294 and 1,437 metabolites were identified in ESI+ and ESI− modes, respectively, after removing low mass ions with relative standard deviation (RSD) ≤30%. Fractional scatter plots of the 2D PLS-DA model showed satisfactory modelling and distinguished the experimental group from the control group in ESI+ mode (Figure 5A,a) and ESI− mode (Figure 5A,b). Validation of the model revealed no overfitting, indicating that the model can accurately characterise the samples and can be used for further data analysis.

Consistently, intestinal metabolites were significantly different between the two groups by VIP criteria (VIP >1) and student’s *t*-test (*p* < 0.05). Heatmaps constructed on the basis of significantly altered metabolites also showed that the separation between the control, SuHx, SuHxA and SuHxM treatment groups basically existed in the ESI+ (Figure 5B,a) and ESI− (Figure 5B,b) modes, where in the ESI+ mode, the SuHx, SuHxA and especially the SuHxA treatment groups were closer to the normal control group, which suggests that SuHxA treatment group has a better therapeutic effect on mice with hypoxia-induced metabolic disorders. However, the relevance of the SuHxM treatment group was closer to the normal control group in the ESI− mode.

In addition, we annotated the identified metabolites using the KEGG database to understand the functional characteristics of the different metabolites and to identify the major biochemical metabolic pathways and signal transduction pathways of the metabolites. As shown in Figure 5C, the number of metabolites in each metabolic pathway is represented in each of the two ionic modes. Amino acid metabolism, cofactor and vitamin metabolism, lipid metabolism, carbohydrate metabolism, nucleotide metabolism, exogenous biodegradation metabolism, biosynthesis of other secondary metabolites, and energy metabolism are the major biochemical metabolic pathways of intestinal metabolites.

A total of 905 differentially expressed metabolites were identified in the serum of the model and control groups in both ESI+ (Figure 5D,a) and ESI− (Figure 5D,b) modes (*p* < 0.05), of which 363 and 542 metabolites rose and fell in the SuHx group. In the SuHx group, the abundance of metabolites such as indole-3-propionic acid, tryptophan, penicillin G, butyric acid and deoxycholic acid decreased and the abundance of harmful metabolites such as monocrotaline, bile acids and cyclophosphamide increased (Table 1).

**Table 1:** Details of differential metabolites by group.

ID	Metabolite	Mode	Con	SuHx	SuHA	SuHM
pos_8323	Indole-3-propionic acid	pos	5.08	3.45	4.85	4.53
pos_7248	Tryptophol	pos	5.25	4.53	5.02	5.2
pos_7691	Penicillin G	pos	5.6	4.92	5.66	5.67
neg_6621	Butyric acid	neg	4.84	4.35	4.63	4.82
neg_18	Deoxycholic acid	neg	7.26	6.72	7.25	7.06
pos_9535	Monocrotaline	pos	5.22	6.18	5.2	5.28
pos_3	Bile acid	pos	6.54	8.04	6.97	6.35
pos_104	Cyclophosphamide	pos	6.41	6.98	6.59	6.53

The differentially expressed metabolites were subjected to enrichment analysis to detect metabolic pathways or enzymes that may be affected. Of the total 230 metabolic pathways enriched in the enrichment analysis, the enrichment results with significant differences were 20 metabolic pathway maps. The results of this analysis showed (Figure 5E) that the significant differences were in the 20 metabolic pathways of Biosynthesis of phenylpropanoids, linoleic acid metabolism, tryptophan metabolism, glycerophospholipid metabolism, phenylpropanoid biosynthesis and phenylalanine metabolism were more enriched (*p* < 0.01).

### Results of combined gut microbiological and metabolite analyses in SuHx mice

**Figure 6:**
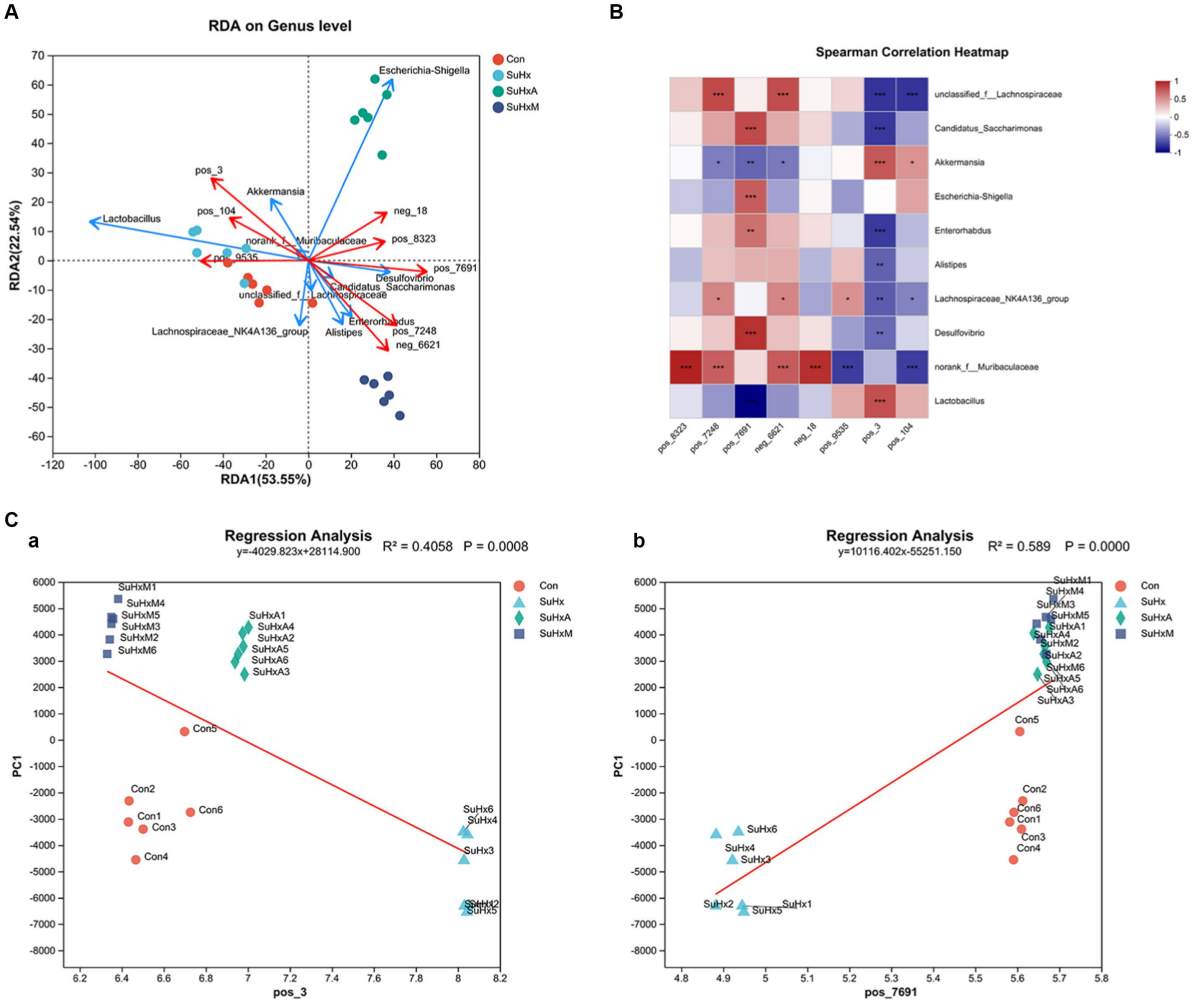
Correlation plots of microbial diversity and metabolome of the gut contents of mice in each group. **(A)** Scatterplot of RDA/CCA analysis of microbial diversity and metabolome of the gut contents of mice in each group. **(B)** Heatmap plot of the correlation between co-expression clusters of the metabolites and the microbiota. **(C)** Sequential regression analysis of the environmental factors between the metabolites penicillin G and bile acids and the microbiota. *N* = 6 in each group.

Finally, we analysed the possible links between gut microbiota and metabolites. In order to visualise the relationship between the flora and environmental factors, we examined the relational relationship between environmental factors, samples, and flora based on microbial diversity by the RDA/CCA sequencing method and correlation Heatmap plots. The results of the RDA/CCA sequencing method showed (Figure 6A) that the deleterious metabolites, such as wild lily alkaloids, bile acids, and cyclic phosphonoacetyls, were associated with additionally *g_norank_f_Muribaculaceae* and *g_Lachnospiraceae_NK4A136_group* were enriched near the samples of SuHx group and had a positive correlation but a negative correlation in the control, SuHxA and SuHxM groups. *Alistipes* were enriched in the control group. In addition, metabolites such as indole-3-propionic acid, tryptophan, penicillin G, butyric acid and deoxycholic acid were enriched with beneficial bacteria such as *g_Enterorhabdus* and *g_Candidatus_Saccharimonas* in the SuHxA and SuHxM groups and had positive correlation but negative correlation in the SuHx group.

On the basis of microbial diversity, at the SuHx level, correlation Heatmap plot results showed (Figure 6B) that *Ackermannia* was positively correlated with the genera bile acids (*r* = 0.66319) 0.03046 and cyclophosphamide (*r* = 0.42783), but negatively correlated with penicillin G (*r* = −0.53043) and butyric acid (*r* = −0.46087). *g_Escherichia-Shigella* was positively correlated with cyclophosphamide (*r* = 0.66319) but negatively correlated with butyric acid (*r* = −0.31929). *g_norank_f_Muribaculaceae* genera were positively correlated with indole-3-propionic acid (*r* = 0.87913), tryptophan (*r* = 0.63478), penicillin G butyric acid (*r* = 0.65391) and deoxycholic acid (*r* = 0.8287) were positively correlated. *g_Candidatus_Saccharimonas* was positively correlated with penicillin G (*r* = 0.72059) but negatively correlated with bile acids (*r* = −0.68421).

Secondly, the magnitude of the effect of this metabolite on the differences in the community composition of the samples was assessed by environmental factor ordination regression analysis, based on the results of the PCA analysis, scatter plots made with each sample and the environmental factor corresponding to that sample and linear regression, which was used to study the correlation between penicillin G (Figure 6C,a) and bile acids (Figure 6C,b) and the microbial flora structure. The results showed that penicillin G among the beneficial metabolites (*r* = 0.589) and bile acids among the deleterious metabolites (*r* = 0.4058) had a high degree of explaining the differences in the samples on the ordination axis.

## Discussion

In the current study, we present novel findings regarding the therapeutic effects of Ang1-7 and MLN-4760 on pathological alterations within the intestinal tract, encompassing changes in microbial diversity of intestinal contents and metabolite profiles, including increased intestinal fibrosis, disorganization of the muscular layer in the intestinal wall, villi hypoplasia and heightened intestinal permeability in SuHx group mice. These observed therapeutic effects hold significant implications for understanding the interactions between the host microbiome and the correlation between intestinal microbes and metabolites in the pathogenesis of SuHx-induced pathology. Marsh et al. (2018) and Wu et al. (2022) stated that the gut microbiota can modulate the immune status of the host, and altered gut microbiota composition and activation of inflammation support the involvement of gut ecological dysregulation in the inflammatory process of PAH. In addition, the HySu-induced group showed a significant increase in the abundance of thick-walled phyla, a significant decrease in the abundance of anamorphic phyla and unidentified bacteria, and an increase in the F/B ratio (Luo et al., 2023). Intestinal villi not only harbor digestive enzymes but also contribute to an enlarged surface area for nutrient absorption. Therefore, the observed impaired intestinal pathology in PH animals may compromise metabolism and nutrient absorption, potentially fostering an environment conducive to the proliferation of pathogenic microbial populations (Sharma et al., 2020a,b).

Prior research has demonstrated that the *norank_f_Muribaculaceae* microbial genera exhibit a significant increase in ulcerative colitis model mice. Moreover, treatment with deferasirox, known for its ability to reduce inflammatory factors and oxidative stress, results in a decrease in *norank_f_Muribaculaceae* genera in ulcerative colitis model mice, accompanied by elevated expression levels of tight junction proteins (Wu et al., 2023). In contrast, our current study revealed a reduction in the relative abundance of the anti-inflammatory microorganism *norank_f_Muribaculaceae* spp. In the SuHx group of mice, with a more pronounced decrease observed in the SuHxA and SuHxM groups. Previous studies have found reduced plasma levels of SCFA and secondary bile acids in PAH patients with a gut microbiome enriched for microbial genes encoding the pro-inflammatory microbial metabolite trimethylamine (Oliveira, 2023). Consistent with previous findings of decreased abundance of beneficial short-chain fatty acid (SCFA)-producing bacteria in the SuHx group, such as the butyrate-producing and propionate-producing bacterial genera such as *g_Candidatus_Saccharimonas*, these alterations may alter the intestinal epithelium and increase intestinal permeability, and thus we suggest that this bacterial genus may alter intestinal epithelial cells and increase intestinal permeability. Previous research has highlighted the critical role of butyrate in anti-inflammatory processes and maintaining the integrity of the intestinal barrier, as evidenced by increased intestinal permeability observed in the SuHx group of mice. Emoto et al. (2016) have reported that hypoxia exposure reduces oxygen pressure in the small intestine, induces alterations in the gut microbiota, sympathetic activation in SuHx mice, increases intestinal permeability, and inflammatory suppression associated with a decrease in *Akkermansia*. In the present study, we found that the establishment of SuHx mouse model and associated with typical intestinal flora, metabolites and pathological alterations, such as reduction of antioxidant flora *Akkermansia* and anti-inflammatory metabolite butyric acid in the intestinal contents of mice in the SuHx group, significant increase in the degree of intestinal fibrosis and thickness of the muscularis propria, and significant reduction in the length of the villi.

Furthermore, alterations in tryptophan metabolites and metabolic enzymes have been documented in patients with inflammatory bowel disease (IBD), where serum and fecal levels of tryptophan are notably lower compared to healthy subjects (Shiomi et al., 2011; Lamas et al., 2016). Building upon these findings, we conducted further investigations into the metabolic profile of intestinal contents. Our results revealed significant reductions in metabolites such as butyric acid and tryptophan in the SuHx group, both of which possess anti-inflammatory properties and contribute to the maintenance of immune cell homeostasis and function. Importantly, after treatment with Ang1-7 and MLN-4760, the levels of these metabolites progressed towards normal levels. Therefore, it can be inferred that alterations in these metabolites exacerbate hypoxia-induced cardiovascular disease and contribute to intestinal damage and disturbances in microbial diversity. In addition to this, the intestines levels of inflammatory cytokines IL-10 and MCP-1 were elevated in the SuHx group of mice in the present study, a result that supports that changes in intestinal microbial metabolites promote perivascular inflammation, and proliferation of pulmonary vascular smooth muscle cells, which triggers lung injury and pulmonary vascular remodeling, and ultimately leads to pulmonary arterial hypertension (Karoor et al., 2021; Song et al., 2021; Huang et al., 2022).

In the current study, the effects of MLN-4760 as an ACE2 inhibitor on gut microbes and metabolites differed from those observed in previous related studies. While prior research has demonstrated the attenuation and arrest of PH progression through various ACE2-related interventions, such as ACE2 supplementation, activation of endogenous ACE2, overexpression via viral-mediated gene delivery, or oral delivery of ACE2 through transplasmid technology (Sharma et al., 2020a,b), our findings regarding MLN-4760 present a distinct outcome. Contrary to expectations, MLN-4760 did not induce changes in the expression of inflammatory genes (TNF, Nos2, Ptgs1, Ptgs2) or the anti-inflammatory gene Pparg. Furthermore, gene expression analyses in our study did not confirm the pro-inflammatory effects of MLN-4760 (Ayuso et al., 2021). Our results were consistent with those of previous studies, indicating that compared with SuHx group, the contents of anti-inflammatory factors IL-10 and IL-12p70 in intestinal tissues of SuHxM group were increased, and the abundance of short-chain fatty acid microorganism *norank_f_Muribaculaceae* in intestinal contents was increased. Increased levels of the anti-inflammatory metabolite *Butyric acid*. Additionally, another study reported an increase in the expression of antioxidant genes Sod1, Sod2, and Hmox1, as well as elevated plasma levels of the antioxidant molecule H_2_S following MLN-4760 treatment. These findings suggest a potential compensatory mechanism for the oxidative damage induced by ACE2 inhibitor-MLN-4760 treatment (Kluknavsky et al., 2022).

The gut microbiota serves as a source of both anti-inflammatory and pro-inflammatory metabolites, including SCFA, which can influence the integrity of the gut barrier. Excessive production of potential toxins by the gut microbiota may compromise the gut barrier, leading to increased circulation of microbial ligands associated with microbe-associated molecular patterns (Moutsoglou et al., 2023). Furthermore, alterations in the composition of gut microbiota metabolites, such as secondary bile acids and trimethylamine, can contribute to systemic immune dysregulation. Considered as another metabolic organ of the host, the gut microbiota and its metabolites play a crucial role in influencing host health (Chung et al., 2018). Metabolic disorders are often associated with changes in the composition and function of the gut microbiota, which interact with the host by metabolizing various exogenous dietary substrates or endogenous host compounds. While Spearman’s correlation analysis revealed certain associations between the gut microbiota and fecal metabolites, establishing a clear causal relationship remains challenging. Notably, our analysis identified correlations between *Akkermansia* spp. and low levels of expression of beneficial metabolites such as indole-3-propionic acid, tryptophan, penicillin G, butyric acid and deoxycholic acid.

In SuHx group, our study highlights significant gut ecological dysregulation associated with Sugen5416/hypoxia-induced disease development. Moreover, we elucidate the therapeutic role of Ang1-7 and MLN-4760 in the progression of this pathology. Specifically, our findings underscore the effects of Ang1-7 and MLN-4760 on gut pathological alterations, as well as alterations in the gut microbiome and metabolome.

These results contribute to a deeper understanding of the intricate relationship between the gut and lung in the SuHx model, shedding light on the underlying mechanisms involving gut microbes and metabolites. Furthermore, our findings offer new insights into potential therapeutic, diagnostic, or management strategies for hypoxia-induced gut pathology. Overall, this study enhances our comprehension of the unique characteristics of gut-lung interactions and provides a foundation for further exploration in this field.

## Data availability statement

The raw data supporting the conclusions of this article will be made available by the authors, without undue reservation.

## Ethics statement

The animal study was approved by Experimental Animal Ethics Committee of the Xinjiang Medical University. The study was conducted in accordance with the local legislation and institutional requirements.

## Author contributions

AsA: Writing – review & editing, Writing – original draft, Data curation. AiA: Writing – review & editing, Writing – original draft, Investigation. TY: Writing – review & editing, Project administration, Conceptualization. LF: Writing – review & editing, Methodology, Investigation. AdA: Writing – review & editing, Visualization, Supervision, Data curation. YZ: Writing – review & editing, Visualization, Software. DS: Writing – review & editing, Methodology, Data curation. YN: Writing – review & editing, Resources, Funding acquisition.

## Footnotes

^1^ https://cloud.majorbio.com
